# Adverse Childhood Experiences and Neurocognition in Schizophrenia Spectrum Disorders: Age at First Exposure and Multiplicity Matter

**DOI:** 10.3389/fpsyt.2021.684099

**Published:** 2021-07-05

**Authors:** Justyna Kasznia, Aleksandra Pytel, Bartłomiej Stańczykiewicz, Jerzy Samochowiec, Joanna Preś, Karolina Rachubińska, Błażej Misiak

**Affiliations:** ^1^Inpatient Psychiatric Unit, Municipal General Hospital, Ostrów Wielkopolski, Poland; ^2^Department of Nervous System Diseases, Wroclaw Medical University, Wroclaw, Poland; ^3^Department of Psychiatry, Pomeranian Medical University, Szczecin, Poland; ^4^Institute of Psychology, University of Szczecin, Szczecin, Poland; ^5^Division of Consultation Psychiatry and Neuroscience, Department of Psychiatry, Wroclaw Medical University, Wroclaw, Poland

**Keywords:** stress, psychosis, childhood trauma, brain development, childhood maltreatment

## Abstract

Adverse childhood experiences (ACEs) might be related to cognitive impairments observed in schizophrenia spectrum disorders (SSD). However, it remains unknown what aspects of ACEs are associated with cognitive impairments in SSD. Therefore, we aimed to investigate the association between various characteristics of ACEs (age at first exposure, severity, and multiplicity) and cognition in SSD and healthy controls (HCs). We enrolled 127 individuals with SSD and 56 HCs. Cognitive performance was assessed using the Repeatable Battery for the Assessment of Neuropsychological Status (RBANS). The Childhood Experience of Care and Abuse Questionnaire was administered to record a history of ACEs. The following characteristics of ACEs were analyzed: multiplicity, severity, and age at first exposure. Individuals with SSD had significantly lower scores on all RBANS domains. Multiplicity and severity of ACEs were significantly higher in patients with SSD compared to HCs. In both groups, greater multiplicity of ACEs was associated with lower scores of global cognition and delayed memory. Additionally, in subjects with SSD, greater multiplicity and younger age at first exposure were associated with lower scores of attention. The present findings indicate that greater multiplicity and younger age at first exposure are the most important aspects of ACEs contributing to cognitive impairments observed in SSD. Moreover, ACEs might exert differential impact on cognition in SSD and HCs.

## Introduction

Convincing evidence indicates that a history of adverse childhood experiences (ACEs), such as emotional abuse or neglect, physical and sexual abuse, increase a risk of schizophrenia spectrum disorders (SSD) (1). It has been estimated that about one third of individuals with psychosis report childhood physical, sexual or emotional abuse (2). Various psychological processes have been recognized to mediate the association between ACEs and psychosis risk, including dissociation, post-traumatic stress disorder symptoms, emotional dysregulation, and negative schemas (3). Moreover, ACEs have been associated with a number of stress-related biological alterations in psychosis (4).

It has been shown that ACEs can also impact clinical expression of SSD. For instance, a recent meta-analysis revealed that ACEs are mainly related to higher severity of hallucinations and delusions (5). Only childhood neglect was found to be correlated with negative symptoms. Additionally, ACEs might be related to unfavorable clinical and functional outcomes of psychosis (6, 7). Another meta-analysis demonstrated that ACEs are associated with worse general cognition and working memory impairments in this clinical population (8). Vargas et al. (8) also tested a number of potential moderators, including age, gender, the use of first-episode psychosis populations and covariates (age, gender, and premorbid IQ). However, none of them was found to correlate with effect size estimates. Moreover, this meta-analysis did not include a number of moderators that were not recorded by eligible studies, including timing, severity and multiplicity of exposure.

Cognitive impairments represent core clinical characteristics of SSD that appear in the premorbid phase and are present in the majority of patients (9, 10). These impairments include deficits across a number of cognitive domains, such as current IQ, category fluency, verbal and working memory, attention and response inhibition (10). Cognitive impairments in SSD can be attributed to various neuroanatomical and electrophysiological alterations, of which, volume deficits in the medial temporal lobe, including the hippocampus, and the prefrontal cortex have been widely reported (11). According to neurodevelopmental considerations, the onset of SSD together with their core clinical characteristics represent the final consequence of various genetic and environmental insults that affect the brain development at different stages (12). However, the consequences of these insults can be deleterious when they act at “critical windows” of the brain development (13).

In light of the neurodevelopmental theory of SSD, considering the effects of ACEs on cognition as a dichotomous insult without detailed recognition of their characteristics might be insufficient to understand the impact of early-life stress. Therefore, in the present study, we investigated whether the extent of cognitive impairments in SSD is associated with such characteristics of ACEs as age at first exposure, multiplicity, and severity. Furthermore, we tested the hypothesis that the impact of these characteristics might be different in subjects with SSD compared to healthy controls.

## Method

### Participants

Inpatients with SSD were recruited at two university hospitals (Department and Clinic of Psychiatry at Wroclaw Medical University, Wroclaw, Poland; Department and Clinic of Psychiatry at Pomeranian Medical University, Szczecin, Poland) and one general hospital (Inpatient Psychiatric Unit, Municipal General Hospital, Ostrów Wielkopolski, Poland) in the years 2016 – 2020 (*n* = 127). Among them, there were 42 inpatients admitted for the first time. This subgroup of participants met the criteria of schizophrenia, schizoaffective disorder, schizophreniform disorder and brief psychotic disorder. In patients who were not admitted for the first time were diagnosed with schizophrenia or schizoaffective disorder. A diagnosis of SSD was based on the DSM-IV criteria, using the Operational Criteria for Psychotic Illness (OPCRIT) checklist (14). A severity of clinical manifestation was recorded using the Positive and Negative Syndrome Scale (PANSS) (15). The majority of them (*n* = 125) were receiving antipsychotic treatment with mean chlorpromazine equivalent dosage (CPZeq) of 357.7 mg/day (SD = 388.7 mg/day).

Healthy controls (*n* = 56) were recruited at Wroclaw Medical University (Wroclaw, Poland) through advertisements. They had absent family history of mood and psychotic disorders in first- and second-degree relatives. The protocol of this study was approved by the Ethics Committee at Wroclaw Medical University, Wroclaw, Poland. All participants provided written informed consent.

### Assessment of Cognitive Performance

The Repeatable Battery for the Assessment of Neuropsychological Status (RBANS) was used to examine cognitive performance (16). The RBANS consists of 12 cognitive tasks grouped into five indexes: (1) immediate memory (list learning and story memory); (2) visuospatial/constructional abilities (figure copy and line orientation); (3) language (picture naming and semantic fluency); (4) attention (digit span and coding) and (5) delayed memory (list recall, list recognition, story memory, and figure recall). Higher scores indicate better cognitive performance.

### Assessment of ACEs

The Childhood Experience of Care and Abuse Questionnaire (CECA.Q) was administered to obtain data on a history of ACEs (17). The CECA.Q is a self-report that records a history of the following ACEs before the age of 17 years: (1) parental loss; (2) mother antipathy; (3) mother neglect; (4) mother psychological abuse; (5) father antipathy; (6) father neglect; (7) father psychological abuse; (8) role reversal; (9) physical abuse and (10) sexual abuse. The subscales for parental psychological abuse and role reversal were not validated against interview, and thus they were excluded from data analysis in the present study.

In our study, three measures of ACEs were analyzed: (1) age at first exposure; (2) multiplicity and (3) severity. Age at first exposure was defined as the age when the first stressful experience appeared. Multiplicity was included as the number of ACEs reported by each participant (range: 0–7). In turn, severity was calculated for all ACEs together, except for parental loss, as the CECA.Q does not include the severity score for this category of ACEs. More specifically, we divided reported severity of exposure by the maximum severity score that can be obtained for specific category of ACEs. Next, all severity scores were summarized and divided by a number of ACEs categories (*n* = 6).

### Data Analysis

Data analyses were carried out using the Statistical Package for Social Sciences, version 20 (SPSS Inc., Chicago, Illinois, USA). Normality of data distribution was assessed using the Kolmogorov-Smirnov test. Bivariate comparisons were performed using the χ^2^ test or the Mann-Whitney *U*-test, where appropriate. Spearman rank correlation coefficients were used to analyze bivariate correlations. The association between the measures of ACEs and cognitive performance was tested using the linear regression analyses. Due to non-normal distribution, the scores of specific RBANS domains were transformed to *z*-scores. Similarly, the measures of ACEs were transformed to *z*-scores to limit potential collinearity. Subsequently, interaction terms between the group status (SSD vs. healthy controls) and the measures of ACEs (*z*-scores) were created. The RBANS *z*-scores were included as a dependent variable. Group status, the measures of ACEs (age at exposure onset, severity, and multiplicity) and interaction terms were included as independent variables. Age and gender were added as covariates. Given that lower educational achievement might be strongly associated with SSD and account for cognitive impairment (18), the number of education years was added as a covariate in a hierarchical manner. The variance inflation factor (VIF) was assessed as the measure of collinearity. The VIF > 4 was considered to indicate significant multicollinearity (19). Linear regression lines were plotted according to the following equation (B refers to unstandardized coefficients): y = B (constant) + B (effect of group status)^*^group status + B (the effect of ACEs measure) + B (interaction term)^*^group status. The group status was dummy coded with the value of “1” assigned to individuals with SSD and the value of “0” assigned to healthy controls. Linear regression lines were plotted for the model that included the number of education years if a significant *R*^2^ change was observed. Otherwise, the plot was prepared for the model without the number of education years. Results were considered statistically significant if the *p*-value was <0.05.

## Results

Main characteristics of the sample are reported in Table 1. There were no significant between-group differences with respect to age (*U* = 3,366.5, *p* = 0.566) and gender (χ^2^ = 0.4, *p* = 0.518). As expected, individuals with SSD had significantly lower education level (*U* = 889.5, *p* < 0.001) and showed worse performance on all RBANS domains (immediate memory: *U* = 690.0, *p* < 0.001; visuospatial/constructional abilities: *U* = 1,073.0, *p* < 0.001; language: *U* = 1,080.0, *p* < 0.001; attention: *U* = 424.0, *p* < 0.001; delayed memory: *U* = 476.0, *p* < 0.001). Individuals with SSD had significantly higher multiplicity (χ^2^ = 10.4, *p* = 0.001) and severity scores (*U* = 4,965.5, *p* < 0.001) of ACEs compared to healthy controls. At least one category of ACEs (χ^2^ = 10.4, *p* = 0.001) as well as a history of mother antipathy (χ^2^ = 7.2, *p* = 0.007), mother neglect (χ^2^ = 9.9, *p* = 0.002), father antipathy (χ^2^ = 7.7, *p* = 0.006), physical abuse (χ^2^ = 6.9, *p* = 0.009), and sexual abuse (χ^2^ = 8.3, *p* = 0.004) were significantly more frequent in subjects with SSD compared to healthy controls. However, both groups did not differ significantly in terms of age at first exposure to ACEs (*U* = 1,037.5, *p* = 0.616).

**Table 1:** General characteristics of the sample.

	**SSD, *n* = 127**	**Controls, *n* = 56**	**Statistics**
Age, years	39.1 ± 13.8	38.3 ± 6.8	*U* = 3,366.5, *p* = 0.566
Gender, males (%)	61 (48.0)	24 (42.8)	*χ^2^* = 0.4, *p* = 0.518
Education, years	13.2 ± 2.8	16.0 ± 2.4	***U*** **=** **889.5**, ***p*** **<** **0.001**
CECA.Q—age at first exposure	9.5 ± 4.6	9.0 ± 4.6	*U* = 1,037.5, *p* = 0.616
CECA.Q—multiplicity	2.3 ± 1.8	1.1 ± 1.3	***U*** **=** **4,940.5**, ***p*** **<** **0.001**
CECA.Q—severity	0.45 ± 0.28	0.34 ± 0.36	***U*** **=** **4,965.5**, ***p*** **<** **0.001**
CECA.Q—multiplicity > 0, *n* (%)	101 (79.5)	32 (57.1)	***χ**^**2**^* **=** **10.4**, ***p*** **=** **0.001**
CECA.Q—parental loss, *n* (%)	38 (29.9)	12 (21.4)	*χ^2^* = 1.5, *p* = 0.223
CECA.Q—mother antipathy, *n* (%)	42 (33.1)	8 (14.3)	***χ**^**2**^* **=** **7.2**, ***p*** **=** **0.007**
CECA.Q—mother neglect, *n* (%)	35 (27.6)	4 (7.1)	***χ**^**2**^* **=** **9.9**, ***p*** **=** **0.002**
CECA.Q—father antipathy, *n* (%)	45 (35.4)	9 (16.1)	***χ**^**2**^* **=** **7.7**, ***p*** **=** **0.006**
CECA.Q—father neglect, *n* (%)	36 (28.3)	15 (26.8)	*χ^2^* = 0.1, *p* = 0.733
CECA.Q—physical abuse, *n* (%)	55 (43.3)	13 (23.2)	***χ**^**2**^* **=** **6.9**, ***p*** **=** **0.009**
CECA.Q—sexual abuse, *n* (%)	29 (22.8)	3 (5.4)	***χ**^**2**^* **=** **8.3**, ***p*** **=** **0.004**
RBANS—immediate memory	37.4 ± 9.9	51.5 ± 6.2	***U*** **=** **690.0**, ***p*** **<** **0.001**
RBANS—visuospatial/constructional abilities	31.7 ± 6.4	37.9 ± 2.3	***U*** **=** **1,073.0**, ***p*** **<** **0.001**
RBANS—language	25.7 ± 5.6	33.6 ± 6.5	***U*** **=** **1,080.0**, ***p*** **<** **0.001**
RBANS—attention	38.2 ± 8.2	68.9 ± 8.9	***U*** **=** **424.0**, ***p*** **<** **0.001**
RBANS—delayed memory	38.4 ± 11.0	55.5 ± 4.7	***U*** **=** **476.0**, ***p*** **<** **0.001**
First admission, *n* (%)	42 (33.1)	-	-
PANSS total score	85.7 ± 30.3	-	-
CPZeq, mg/day	357.7 ± 388.7	-	-

*CECA.Q, the Childhood Experience of Care and Abuse Questionnaire; CPZeq, chlorpromazine equivalent dosage; PANSS, the Positive and Negative Syndrome Scale; RBANS, the Repeatable Battery for the Assessment of Neuropsychological Status; SSD, schizophrenia spectrum disorders*.

*Significant differences (p < 0.05) were marked with bold characters*.

Bivariate correlations between the measures of ACEs and the RBANS scores are shown in Table 2. Younger age at first exposure was associated with significantly lower RBANS scores (visuospatial/constructional abilities: *r* = 0.338, *p* < 0.01; language: *r* = 0.243, *p* < 0.05; attention: *r* = 0.426, *p* < 0.001; delayed memory: *r* = 0.357, *p* < 0.01; global cognition: *r* = 0.435, *p* < 0.001), except for the score of immediate memory (*r* = 0.222, *p* > 0.05) in subjects with SSD but not in healthy controls (immediate memory: *r* = 0.126, *p* > 0.05; visuospatial/constructional abilities: *r* = 0.133, *p* > 0.05; language: *r* = 0.198, *p* > 0.05; attention: *r* = 0.014, *p* > 0.05; delayed memory: *r* = −0.102, *p* > 0.05; global cognition: *r* = −0.127, *p* > 0.05). Greater multiplicity of ACEs was also related to significantly lower RBANS scores (visuospatial/constructional abilities: *r* = −0.271, *p* < 0.01; attention: *r* = −0.301, *p* < 0.01; delayed memory: *r* = −0.293, *p* < 0.01 and global cognition: *r* = −0.302, *p* < 0.01 in subjects with SSD as well as immediate memory: *r* = −0.336, *p* < 0.05; language: *r* = −0.271, *p* < 0.05; delayed memory: *r* = −0.307, *p* < 0.05, and global cognition: *r* = −0.316, *p* < 0.01 in healthy controls). There was a significant negative correlation between overall severity of ACEs and the score of attention in healthy controls (*r* = −0.282, *p* < 0.05). In both groups of participants, overall severity and multiplicity of ACEs were significantly and positively correlated (SSD: *r* = 0.772, *p* < 0.001, healthy controls: *r* = 0.810, *p* < 0.001).

**Table 2:** Bivariate correlations between the measures of ACEs and cognitive performance scores.

**Group**	**Variable**	**1**.	**2**.	**3**.	**4**.	**5**.	**6**.	**7**.	**8**.
SSD	1. ACEs—age at first exposure	-							
	2. ACEs—multiplicity	*r* = −0.230	-						
	3. ACEs—severity	*r* = −0.047	*r* = 0.772^c^	-					
	4. Immediate memory	*r* = 0.222	*r* = −0.183	*r* = −0.038	-				
	5. Visuospatial/constructional	*r* = 0.338^b^	*r* = −0.271^b^	*r* = −0.134	*r* = 0.568^c^	-			
	6. Language	*r* = 0.243^a^	*r* = −0.190	*r* = −0.035	*r* = 0.623^c^	*r* = 0.418^c^	-		
	7. Attention	*r* = 0.426^c^	*r* = −0.301^b^	*r* = −0.108	*r* = 0.643^c^	*r* = 0.661^c^	*r* = 0.539^c^	-	
	8. Delayed memory	*r* = 0.357^b^	*r* = −0.293^b^	*r* = −0.156	*r* = 0.740^c^	*r* = 0.594^c^	*r* = 0.596^c^	*r* = 0.626^c^	-
	9. Global cognition	*r* = 0.435^c^	*r* = −0.302^b^	*r* = −0.121	*r* = 0.851^c^	*r* = 0.745^c^	*r* = 0.724^c^	*r* = 0.880^c^	*r* = 0.626^c^
HCs	1. ACEs—age at first exposure	-							
	2. ACEs—multiplicity	*r* = −0.175	-						
	3. ACEs—severity	*r* = −0.253	*r* = 0.810^c^	-					
	4. Immediate memory	*r* = 0.126	*r* = −0.336^a^	*r* = −0.235	-				
	5. Visuospatial/constructional	*r* = 0.133	*r* = −0.120	*r* = 0.001	*r* = 0.195	-			
	6. Language	*r* = 0.198	*r* = −0.271^a^	*r* = −0.134	*r* = 0.368^b^	*r* = 0.250	-		
	7. Attention	*r* = 0.014	*r* = −0.183	*r* = −0.282^a^	*r* = 0.444^b^	*r* = 0.444^b^	*r* = 0.198	-	
	8. Delayed memory	*r* = −0.102	*r* = −0.307^a^	*r* = −0.234	*r* = 0.673^c^	*r* = 0.298^a^	*r* = 0.298^a^	*r* = 0.335^a^	-
	9. Global cognition	*r* = −0.127	*r* = −0.316^b^	*r* = −0.216	*r* = 0.767^c^	*r* = 0.387^b^	*r* = 0.640^c^	*r* = 0.741^c^	*r* = 0.749^c^

*ACEs, adverse childhood experiences; HCs, healthy controls; SSD, schizophrenia spectrum disorders*.

a *p < 0.05*.

b *p < 0.01*.

c *p < 0.001*.

**Figure 1:**
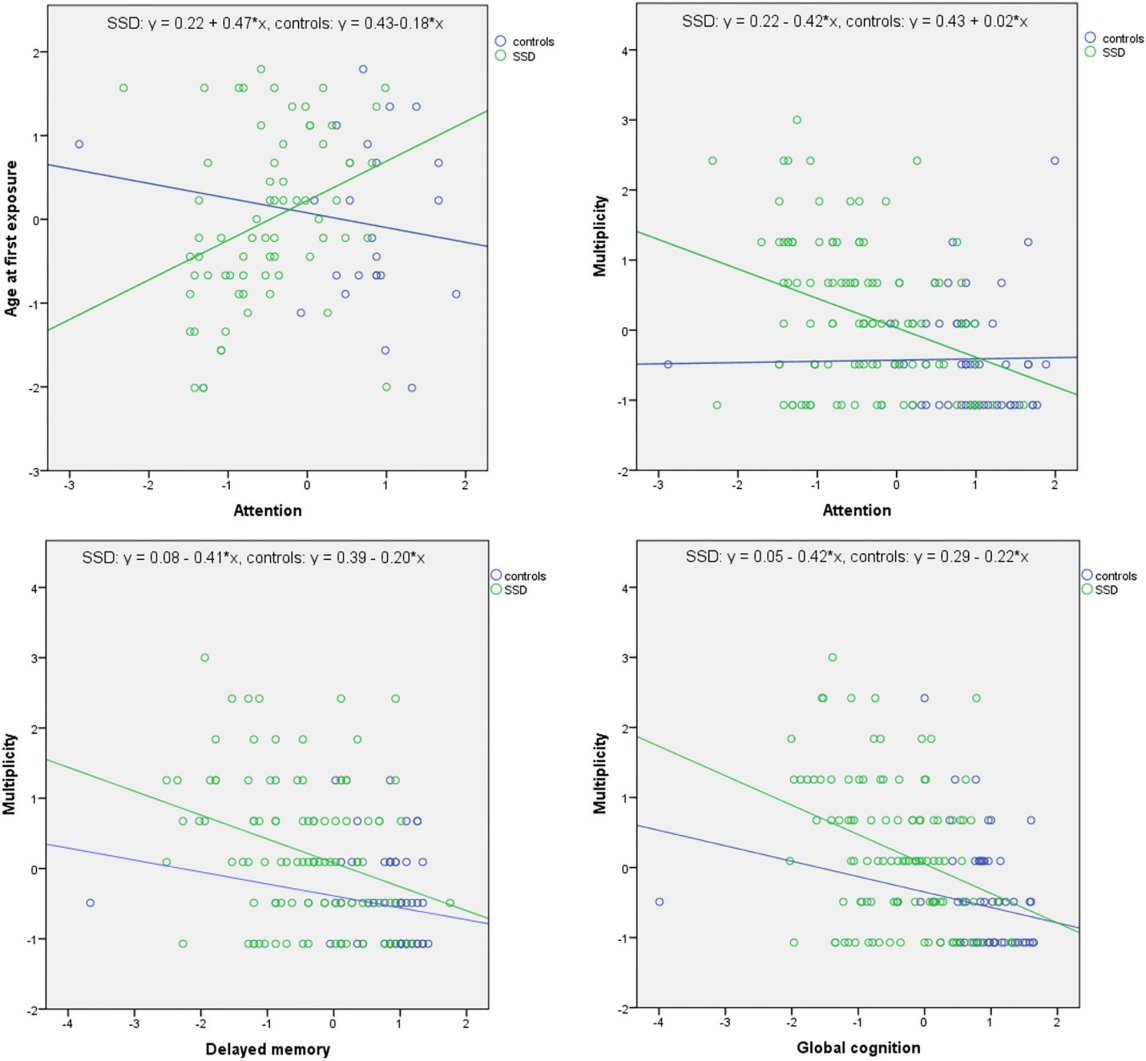
Significant associations between the measures of ACEs and cognition in linear regression analyses. Data expressed as *z*-scores. There were significant main effects of multiplicity of ACEs on delayed memory (*B* = −0.201, *p* = 0.040) and global cognition scores (*B* = −0.220, *p* = 0.040). Additionally, significant effects of interactions between group and age at first exposure (*B* = 0.650, *p* = 0.018) as well as between group and multiplicity (*B* = −0.440, *p* = 0.017) on attention scores were found. More specifically, younger age at first exposure and greater multiplicity of ACEs were associated with worse performance of attention in subjects with SSD but not in healthy controls.

Results of linear regression analyses controlling for the effects of age, the number of education years and gender are presented in Table 3. Significant main and interaction effects are shown in Figure 1. There were significant main effects of multiplicity of ACEs on delayed memory (*B* = −0.201, *p* = 0.040) and global cognition scores (*B* = −0.187, *p* = 0.031), even if the number of education years was added to independent variables. Additionally, significant effects of interactions between group and age at first exposure (*B* = 0.650, *p* = 0.018) as well as between group and multiplicity (*B* = −0.440, *p* = 0.017) on attention scores were observed. More specifically, younger age at first exposure and greater multiplicity of ACEs were associated with worse performance of attention in subjects with SSD but not in healthy controls, after controlling for the effects of age, gender, and the number of education years.

**Table 3:** Results of linear regression analyses.

	**Independent variable**	**Immediate memory**	**Visuospatial/** **constructional**	**Language**	**Attention**	**Delayed** **memory**	**Global** **cognition**	**VIF**
Model 1	Age	*B* = −0.014, *p* = 0.305	***B*** **=** **−0.033**, ***p*** **=** **0.018**	*B* = −0.024, *p* = 0.097	***B*** **=** **−0.022**, ***p*** **=** **0.009**	*B* = −0.012, *p* = 0.278	***B*** **=** **−0.021**, ***p*** **=** **0.040**	1.726
	Gender	*B* = 0.459, *p* = 0.085	*B* = 0.057, *p* = 0.915	***B*** **=** **0.782**, ***p*** **=** **0.010**	*B* = 0.238, *p* = 0.128	*B* = 0.298, *p* = 0.206	*B* = 0.377, *p* = 0.078	1.113
	Group	***B*** **=** **−0.724**, ***p*** **=** **0.014**	*B* = −0.224, *p* = 0.543	***B*** **=** **−0.697**, ***p*** **=** **0.016**	***B*** **=** **−0.310** ***p*** **<** **0.001**	***B*** **=** **−0.311**, ***p*** **=** **0.020**	***B*** **=** **−0.341**, ***p*** **=** **0.004**	1.419
	Age at first exposure	*B* = −0.078, *p* = 0.565	*B* = −0.048, *p* = 0.661	*B* = −0.191, *p* = 0.213	*B* = −0.190, *p* = 0.566	*B* = −0.068, *p* = 0.376	*B* = −0.120, *p* = 0.384	2.984
	Multiplicity	*B* = 0.311, *p* = 0.162	*B* = 0.523, *p* = 0.785	*B* = −0.405, *p* = 0.176	*B* = 0.046, *p* = 0.387	***B*** **=** **−0.201**, ***p*** **=** **0.040**	***B*** **=** **−0.187**, ***p*** **=** **0.031**	3.597
	Severity	*B* = 0.026, *p* = 0.560	*B* = 0.048, *p* = 0.759	*B* = −0.087, *p* = 0.724	*B* = −0.066, *p* = 0.377	*B* = 0.037, *p* = 0.744	*B* = −0.015, *p* = 0.906	2.257
	Group × age at first exposure	*B* = 0.304, *p* = 0.068	*B* = −0.038, *p* = 0.753	*B* = 0.188, *p* = 0.353	***B*** **=** **0.613**, ***p*** **=** **0.024**	*B* = 0.157, *p* = 0.529	*B* = 0.114, *p* = 0.164	4.000
	Group × multiplicity	*B* = −0.274, *p* = 0.772	*B* = −0.741, *p* = 0.438	*B* = 0.412, *p* = 0.281	***B*** **=** **−0.340**, ***p*** **=** **0.019**	*B* = −0.210, *p* = 0.937	*B* = −0.113, *p* = 0.981	3.874
	Group × severity	*B* = 0.045, *p* = 0.803	*B* = 0.114, *p* = 0.759	*B* = 0.034, *p* = 0.981	*B* = 0.074, *p* = 0.497	*B* = 0.045, *p* = 0.800	*B* = 0.166, *p* = 0.664	2.325
	*R*^2^	0.295	0.225	0.359	0.594	0.361	0.435	-
	*R*^2^ change (*p*)	**0.295 (0.012)**	**0.225 (0.080)**	**0.359 (0.001)**	**0.594 (<** **0.001)**	**0.361 (0.001)**	**0.435 (<** **0.001)**	-
Model 2	Age	*B* = −0.015, *p* = 0.233	*B* = −0.036, *p* = 0.009	*B* = −0.026, *p* = 0.064	***B*** **=** **−0.024**, ***p*** **=** **0.003**	*B* = −0.013, *p* = 0.253	*B* = −0.023, *p* = 0.021	1.745
	Education years	*B* = 0.076, *p* = 0.121	***B*** **=** **0.103**, ***p*** **=** **0.043**	*B* = 0.101, *p* = 0.084	***B*** **=** **0.077**, ***p*** **=** **0.008**	*B* = 0.030, *p* = 0.501	***B*** **=** **0.079**, ***p*** **=** **0.040**	1.283
	Gender	*B* = 0.404, *p* = 0.120	*B* = −0.018, *p* = 0.897	***B*** **=** **0.709**, ***p*** **=** **0.015**	*B* = 0.183, *p* = 0.199	*B* = 0.276, *p* = 0.241	*B* = 0.320, *p* = 0.117	1.128
	Group	*B* = −0.560, *p* = 0.077	*B* = 0.001, *p* = 0.864	*B* = −0.480, *p* = 0.092	***B*** **=** **−0.210**, ***p*** **=** **0.009**	*B* = −0.567, *p* = 0.054	***B*** **=** **−0.242**, ***p*** **=** **0.037**	1.638
	Age at first exposure	*B* = −0.085, *p* = 0.551	*B* = −0.044, *p* = 0.640	*B* = −0.200, *p* = 0.200	*B* = −0.180, *p* = 0.529	*B* = −0.158, *p* = 0.376	*B* = −0.127, *p* = 0.362	2.985
	Multiplicity	*B* = 0.353, *p* = 0.932	*B* = 0.580, *p* = 0.500	*B* = −0.461, *p* = 0.310	*B* = 0.020, *p* = 0.155	***B*** **=** **−0.238**, ***p*** **=** **0.038**	***B*** **=** **−0.220,** ***p*** **=** **0.044**	3.739
	Severity	*B* = 0.027, *p* = 0.832	*B* = 0.051, *p* = 0.794	*B* = −0.085, *p* = 0.686	*B* = −0.064, *p* = 0.316	*B* = 0.022, *p* = 0.443	*B* = −0.013, *p* = 0.860	2.258
	Group × age at first exposure	*B* = 0.308, *p* = 0.239	*B* = 0.044, *p* = 0.749	*B* = 0.200, *p* = 0.346	***B*** **=** **0.650**, ***p*** **=** **0.018**	*B* = 0.158, *p* = 0.532	*B* = 0.129, *p* = 0.153	4.000
	Group × multiplicity	*B* = −0.299, *p* = 0.932	*B* = −0.775, *p* = 0.293	*B* = 0.445, *p* = 0.387	***B*** **=** **−0.440**, ***p*** **=** **0.017**	*B* = 0.277, *p* = 0.957	*B* = −0.200, *p* = 0.702	3.912
	Group × severity	*B* = 0.010, *p* = 0.832	*B* = 0.039, *p* = 0.790	*B* = −0.040, *p* = 0.795	*B* = 0.018, *p* = 0.792	*B* = 0.022, *p* = 0.889	*B* = 0.113, *p* = 0.918	2.388
	*R*^2^	0.325	0.280	0.393	0.643	0.367	0.476	-
	*R*^2^ change (*p*)	0.030 (0.121)	**0.055 (0.043)**	0.034 (0.084)	**0.049 (0.008)**	0.006 (0.501)	**0.041 (0.040)**	-

*Significant results (p < 0.05) were marked with bold characters*.

## Discussion

Findings from the present study imply that ACEs might contribute to cognitive impairments observed in patients with SSD. Notably, we found that greater multiplicity of ACEs might be associated with impairments of delayed memory and global cognition in both groups of participants—individuals with SSD and healthy controls. However, the association between characteristics of ACEs (age at first exposure and multiplicity) and attention scores was found only in patients with SSD. No significant associations with cognition were found for the overall severity of ACEs.

It is important to note that the RBANS attention index is composed of scores from two cognitive tasks (digit span and digit coding) that also enable to assess cognitive domains other than attention. The score of digit span reflects working memory performance, while the digit coding task enables to record processing speed. Impairments of working memory have been widely reported in patients with SSD, also at the onset of psychosis, and may reflect neurostructural and neurofunctional alterations of the prefrontal cortex (20–22). Similarly, impairments measured by the digit coding are already present in individuals at risk of psychosis, those with first-episode psychosis and subjects with chronic schizophrenia, with poor response to treatment (23–25). Our findings are also in line with those obtained by previous studies, including most recent meta-analysis (8). As similar to the present study, Schalinski et al. (26) aimed to examine the association between various aspects of ACEs (duration, multiplicity and severity) and cognitive performance in psychosis. The authors found that abuse at the age of 3 years might be related to impairments of attention, learning and working memory. Additionally, neglect at the age of 3 years was associated with worse performance of attention. No significant associations of duration and multiplicity with neurocognition were reported. However, greater multiplicity and neglect experienced at the age of 11–12 years were associated with worse performance of social cognition. In turn, Li et al. (27) found that a history of various ACEs is associated with lower RBANS scores of language, attention and delayed memory. Other studies, although without insights into detailed characteristics of ACEs, also reported that ACEs are associated with impairments of attention and working memory in psychosis (28–30).

Previous studies did not investigate a differential impact of ACEs on cognition in subjects with psychosis and healthy controls. Therefore, it is difficult to establish unequivocal conclusion whether ACEs differentially impact cognition in these populations. Nevertheless, in both groups of participants, multiplicity of ACEs was correlated with cognition. Similarly, in both groups, multiplicity of ACEs was associated with worse performance of delayed memory and global cognition. Although the present study demonstrated that age at first exposure was similar in both groups of participants, severity and multiplicity scores were significantly higher in subjects with SSD compared to healthy controls. This observation might explain worse cognitive performance in patients with SSD compared to healthy controls, taking into account previous reports that ACEs increase a risk of psychosis with a dose-response effect (31). However, it is still unclear whether specific characteristics of ACEs exert qualitatively differential impact on cognition in subjects with SSD and healthy controls. Moreover, it is warranted to investigate whether neurocognitive deficits attributable to ACEs contribute to other psychopathological symptoms of psychosis. Indeed, there is evidence that there are several cognitive mediators of the association between ACEs and psychopathology. These include, i.e., cognitive styles, negative core/internalized beliefs, negative attributions, evaluating and pathogenic beliefs and early maladaptive schemas (32).

The present findings should also be referred to potential neurobiological mechanisms that may explain the relationship between ACEs and cognition. The hypothalamic-pituitary-adrenal (HPA) axis serves as one of main biological systems responsible for stress response by releasing glucocorticoids. Notably, the hippocampus and prefrontal cortex contain high density of glucocorticoid receptors. These brain regions are responsible for learning and memory processes. Prolonged exposure to glucocorticoids may lead to reduced neurogenesis and synaptic plasticity in the hippocampus and prefrontal cortex (33, 34). Previous meta-analyses have revealed that patients with psychosis show dysfunction of the HPA axis in terms of elevated blood cortisol levels (35), attenuated cortisol awakening response (36) and blunted cortisol response to social stress (37). Moreover, there is evidence that ACEs might contribute to dysfunction of the HPA axis in psychosis (38–41). Our group has recently reported that elevated cortisol levels might be associated with deficits of delayed memory in subjects with SSD (42). In turn, Aas et al. (40) found that elevated hair cortisol levels are correlated with working memory deficits in individuals with schizophrenia and bipolar disorder.

Certain limitations of the present study need to be discussed. First, our sample size, especially with respect to healthy controls, was not large. The difference in sample sizes between individuals with psychotic disorders and healthy controls might also account for observed differences in the association between ACEs and cognition in these groups of participants. However, previous studies in this field were based on similar or even smaller sample sizes. Second, a recall bias should always be taken into consideration when interpreting the data from self-reports of ACEs. This might be of particular importance to ACEs that appear very early in the developmental period. Nevertheless, sufficient test-retest reliability and consistency have been demonstrated for self-reports of ACEs in subjects with psychosis (43, 44). Another point is that the proportion of variance in cognitive performance explained by our linear regression analyses (22.5–64.3%) suggest that other factors might also contribute to cognitive performance, and were not explored in the present study. These might include duration of illness, factors related to overweight or obesity, medication effects and substance use.

In summary, our findings imply that greater multiplicity of ACEs might account for impairments of attention, delayed memory and global cognition, while earlier age of first exposure to ACEs might additionally contribute to impaired attention in subjects with SSD. The impact of ACEs on cognition in individuals with SSD and healthy controls might share some similarities, with multiplicity being the most important factor. These findings provide new support for the neurodevelopmental theory of schizophrenia. A differential impact on specific cognitive domains in individuals with SSD and healthy controls requires additional studies in larger samples. Moreover, the mechanisms underlying the relationship between ACEs and cognitive deficits in SSD also need to be established. Finally, future studies should further investigate whether cognitive impairments attributable to ACEs further shape specific symptoms of psychosis.

## Data Availability Statement

The raw data supporting the conclusions of this article will be made available by the authors, without undue reservation.

## Ethics Statement

The studies involving human participants were reviewed and approved by Ethics Committee at Wroclaw Medical University, Poland. The patients/participants provided their written informed consent to participate in this study.

## Author Contributions

JK: study design, recruitment, and manuscript writing. AP: data collection and manuscript editing. BS: recruitment and manuscript editing. JS: manuscript writing. JP: recruitment and manuscript writing. KR: recruitment and data analysis. BM: recruitment, data analysis, and manuscript writing. All authors contributed to the article and approved the submitted version.

## Conflict of Interest

The authors declare that the research was conducted in the absence of any commercial or financial relationships that could be construed as a potential conflict of interest.
